# A data-driven supervised machine learning approach to estimating global ambient air pollution concentrations with associated prediction intervals

**DOI:** 10.1098/rsos.241288

**Published:** 2025-07-23

**Authors:** Liam Jordan Berrisford, Hugo Barbosa, Ronaldo Menezes

**Affiliations:** ^1^ Department of Mathematics, University of Exeter, Exeter, UK; ^2^ UKRI Centre for Doctoral Training in Environmental Intelligence, University of Exeter, Exeter, UK; ^3^ Department of Computer Science, University of Exeter, Exeter, UK; ^4^ BioComplex Laboratory, Department of Computer Science, University of Exeter, Exeter, UK; ^5^ UK Department of Computer Science, Federal University of Ceará, Fortaleza, Brazil

**Keywords:** machine learning, global air pollution, temporal and spatial modelling, prediction intervals

## Abstract

Global ambient air pollution, a transboundary challenge, is typically addressed through interventions relying on data from spatially sparse and heterogeneously placed monitoring stations. These stations often encounter temporal data gaps due to issues such as power outages. In response, we have developed a scalable, data-driven, supervised machine learning framework. The models produced by the framework are designed to impute missing temporal and spatial measurements, thereby generating a comprehensive dataset for air pollutants including NO_2_, O_3_, PM_10_, PM_2_._5_ and SO_2_. In this work, we produce models providing concentration estimations at 261 377 locations across the globe. The dataset, with a fine granularity of 0.25° spatial resolution at hourly time intervals and accompanied by prediction intervals for each estimate, caters to a wide range of stakeholders relying on outdoor air pollution data for downstream assessments. This enables more detailed studies. Additionally, the model’s performance across various geographical locations is examined, providing insights and recommendations for strategic placement of future monitoring stations to further enhance the model’s accuracy.

## 1. Introduction

Air pollution represents a significant global challenge. Astonishingly, 99% of the global population is exposed to air pollution that exceeds the air quality limits established by the World Health Organization (WHO) [1]. In response, numerous countries have implemented air pollution monitoring systems.^1^ While direct monitoring through stations is an essential initial step in comprehending air pollution, it is impractical to deploy a monitoring station at every location. This impracticality stems from both logistical challenges, such as the necessity for infrastructure like power lines and data connections, and financial considerations, with a standard monitoring station in the UK costing up to £198 000 [2]. Consequently, models are crucial for bridging spatial and temporal gaps in air pollution concentration measurements. Air pollution models, like those used by the WHO, typically provide data with an annual temporal resolution. However, there is an evident gap in global models that focus on hourly air pollution concentrations. Considering that certain air pollution guidelines, including WHO’s, specifically demand hourly resolution [3], the need for comprehensive, hourly resolved air pollution concentration estimates is paramount for effective decision-making. This paper introduces a data-driven, supervised machine learning model framework designed to predict air pollution concentrations at an hourly resolution on a global scale. A single model is created for each air pollutant. There are four key outputs from this work:

(1) Development of a comprehensive air pollution concentration map, covering the entire globe for the year 2022 at 261 377 locations, at an hourly resolution. This map includes concentrations of NO_2_, O_3_, PM_10_, PM_2.5_ and SO_2_, the quintet of pollutants that constitute the Daily Air Quality Index (DAQI) in the UK [4].
(2) Analysis to gauge the feasibility of extrapolating air pollution concentrations from one region to another. This involves addressing the critical question**:** *To what extent can the air pollution data of one country accurately predict the air pollution levels in another?*
(3) Provision of strategic recommendations for the placement of future air pollution monitoring stations, informed by the uncertainty metrics derived from our model.
(4) A comprehensive evaluation of global air quality, identifying regions with the most severe air pollution and pinpointing which of the five DAQI pollutants predominantly contributes to poor air quality in these areas.

The complete spatial map of air pollution concentrations can be used downstream in other scientific studies, such as the health implication of particulate matter (PM) [5], or the impact of ozone on vegetation [6] at a resolution not previously possible. The unprecedented resolution of this map enables more detailed and accurate analyses than were previously feasible. Furthermore, our approach to estimating air pollution concentrations from one country based on data from another represents a step towards open data initiatives. This fosters international data sharing, contributing to a more equitable global understanding of air pollution and research [7]. Our findings illustrate that even data-rich countries like the United Kingdom can benefit from exchanging data with countries that have less comprehensive data. The manuscript explores the concept that air pollution data from France can offer insights into air pollution in the UK, suggesting that international cooperation can be a cost-effective strategy for achieving a complete and detailed spatial air pollution map.

## 2. Related work

There are numerous advantages to estimating air pollution concentrations on a global rather than regional scale. Air pollution is inherently transboundary, disregarding national boundaries. Pollutants released in one country can traverse long distances, impacting areas far from their original emission points, such as Saharan dust adversely affecting air quality in the UK [8]. Additionally, climate change is anticipated to broadly affect global processes that influence air pollution. For example, ozone formation is expected to increase with rising temperatures [9], wildfires are predicted to become more frequent [10], and their contribution to air pollution is likely to grow [11]. Beyond the direct sources of air pollution, climate change is forecasted to alter several influencing factors, such as an increase in stagnation events—periods of minimal wind leading to localized pollution accumulation [12]. Expected changes in precipitation patterns [13] will further complicate matters, affecting the wet deposition process crucial for removing atmospheric pollutants. A comprehensive, spatially complete view of air pollution concentrations globally, at a high temporal resolution like hourly, is essential for understanding the effects of such events. This understanding enables the development of mitigation strategies aimed at improving human and environmental health amid the global air pollution crisis.

### 2.1. Measuring air pollution

The primary method of determining a location’s air pollution concentration is to conduct measurements with specialized equipment. *In situ* measuring equipment can be split into two categories: high-quality stationary monitoring stations, such as those used in the UK Automatic Urban and Rural (AURN) network,^2^ and mobile, low-cost air quality sensors [14], such as Purple Air sensors.^3^ While monitoring stations used in the AURN are desirable, at an individual cost of £198 000 [2], they are impossible to use for complete spatial coverage over a geographic region such as the UK, with the UK only currently having 171 monitoring stations online. This fundamental shortcoming of stationary monitoring stations can be overcome using low-cost sensors, providing measurements over a larger geographic region. However, issues are present with the current state of the technology used in low-cost sensors. Changes in atmospheric composition and meteorological conditions can influence the measurements being made, alongside other air pollutants impacting the measurement of a target pollutant [15]. One of the main costs associated with stationary monitoring stations is the quality control conducted on the sensor, ensuring measurements are made under the same conditions concerning elements, such as the height at which the measurement is taken [16]. Therefore, while low-cost sensors provide a benefit, they should be used cautiously, with their primary purpose being raising awareness rather than applications requiring higher accuracy, such as epidemiological studies or compliance with air quality legislation [17].

*Ex situ* measurements of air pollution concentrations are also possible with remote sensing. Sentinel 5P [18] is a European Space Agency (ESA) satellite providing air pollution concentration measurements over large geographic regions. Sentinel 5P has a near-polar, sun-synchronous orbit [19], meaning that the platform will always pass over a region at a similar time each day. While this makes it great for comparing locations between each other, it makes it challenging to have a neat, complete air pollution concentration picture across a whole day, with questions such as the difference between rush hour and midnight air pollution concentrations impossible to answer. Furthermore, environmental conditions can make measurements at given locations and times impossible [20]. These issues result in the datasets produced by Sentinel 5P being incomplete, with considerable missing data. The recently operational Tropospheric Emissions: Monitoring of Pollution (TEMPO) [21] remote sensing platform has similar properties and limitations to Sentinel 5P, with the critical distinction between the two being TEMPO’s mission of hourly observations over just North America.

While various methods exist for measuring air pollution concentrations, each approach has its limitations in certain aspects. To compensate for these shortcomings, model outputs are employed to augment observational data. This ensures the availability of air pollution concentration maps that are both temporally and spatially complete, facilitating their use in downstream applications.

### 2.2. Modelling air pollution

There are two primary frameworks for modelling air pollution: Lagrangian and Eulerian. Lagrangian models focus on tracking individual air parcels (or particles), aiming to trace their movement through the atmosphere over time and space [22]. By contrast, Eulerian models do not track air parcels individually; instead, they divide the atmosphere into a grid of regions to monitor air pollution concentrations at fixed points over time [23]. Eulerian models are particularly useful for examining the spatial distribution of air pollution across large areas, such as providing a comprehensive view of air pollution throughout Europe [24]. Conversely, Lagrangian models are more appropriate for investigating specific pollution sources, like the dispersion of ash from a volcanic eruption [25]. The research presented in this manuscript utilizes the Eulerian modelling approach, with the goal of employing machine learning techniques to estimate air pollution concentrations on a global scale.

The options for employing a Eulerian air pollution model framework capable of providing global measurements are notably more restricted than those for regional or local models, yet they do exist. GEOS-Chem [26], a mechanistic model, represents a global three-dimensional atmospheric chemistry model that incorporates input data including meteorological factors. However, GEOS-Chem demands significant expertise in the domain to effectively navigate its complexities, alongside substantial infrastructure support. For instance, a standard simulation with GEOS-Chem at 4.00° × 5.00° resolution necessitates 15 GB of RAM.^4^ Should the computational demands of models like GEOS-Chem prove too onerous for certain applications, statistical models, such as land use regression (LUR), may offer a more feasible alternative. LUR provides a method to develop stochastic air pollution models using input data variables like meteorological conditions, terrain, land use and road network data [27].

A rapidly emerging area is the use of deterministic models to address the current gap within the existing suite of models that can provide high-resolution air pollution concentration data, both temporally and spatially, to empower stakeholders to make informed decisions concerning air pollution. A range of these models are based on a data-driven supervised machine learning model where a target vector, generally air pollution concentrations, is estimated from a feature vector, such as meteorological variables. The model aims to learn the relationship between the target and feature vectors in situations where both are available, enabling subsequent predictions of target vectors in situations where only the feature vector is available. In the scientific literature, several studies use machine learning techniques to forecast air pollution concentrations [28–30]. However, this has the limitation of needing existing air pollution data where the forecast is being conducted. Historical air pollution concentrations limit the model’s use to locations where an air pollution monitoring station exists.

Existing studies have tackled the problem of estimating air pollution concentrations in locations without monitoring stations. However, the studies focus either on small geographical areas, such as the Bay of Algeciras (Spain) with hourly temporal resolution [31] or a large geographical area with low temporal resolution, such as monthly [32]. Some work exists in the middle of existing studies, such as daily temporal resolution over a larger spatial area [33,34], or larger area still with annual temporal resolution [35].

In our previous research, we introduced a model that integrates various aspects to predict air pollution concentrations with an hourly temporal resolution across England’s extensive geographical area [36]. This task presented a notable challenge due to the variability of air pollution concentrations across the locations covered. We successfully developed an air pollution model for England, providing data at a 1 km^2^ hourly resolution, which is invaluable for numerous downstream applications. This model employed a modified Eulerian framework, utilizing a machine learning model as a synthetic monitoring station. The model’s process involves simulating the air pollution concentration reading of a monitoring station under the environmental conditions specified by the input data. By training on data that illustrate the relationship between environmental conditions and air pollution concentrations, the model can utilize known environmental conditions across the study area to predict air pollution concentrations in locations where data are less readily available, thus offering a comprehensive overview of air pollution levels. A key advantage of this approach over other deterministic methods is the significant improvement in computational speed, especially critical for predictions at a global spatial resolution. This efficiency gain is achieved by eliminating spatial dependencies between grid cells—treating each synthetic monitoring station as independent, which facilitates computational speed through the parallelization of predictions. This also enables easier data exploration by allowing predictions for individual locations. In this work, we aim to apply this approach globally, a problem that has not yet been tackled, exploring the challenges associated with employing a transboundary data-driven supervised machine learning air pollution model and underscoring the advantages of an ambient air pollution model that scales linearly with computational complexity.

## 3. Data

### 3.1. Target vector: air pollution concentrations

**Figure 1.**
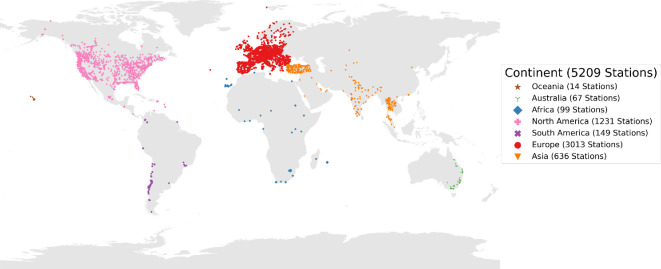
Spatial distribution of monitoring station locations within the OpenAQ dataset for all air pollutants in 2022. The map reveals a high density of stations within Europe and the US relative to the geographical areas these regions cover. This underscores the disparity between countries regarding the extent of their monitoring station networks and the availability of data to address air pollution challenges.

The air pollution data utilized in this study were sourced from OpenAQ [37]. OpenAQ serves as a platform aggregating data from diverse sources into a unified standard format. Given the wide variety of monitoring station networks globally, each operated by different governments and each with its unique data format, amalgamating these data into a consistent format necessary for this research would entail significant effort and data pre-processing from their original datasets. The OpenAQ air pollution dataset is global, offering data from monitoring stations located in various regions, as illustrated in figure 1.

One of the issues with the data acquired was that some of the monitoring stations, rather than measuring in µg m^−3^, would measure in parts per billion (ppb). µg m^−3^ measurements can be calculated from ppb measurements when the molecular weight, temperature and pressure are known [38]. However, this approach introduces another dimension to the study and potentially systematic errors, arising from the fundamental differences in how concentrations are measured across various monitoring networks. As such, we decided that for this study, OpenAQ monitoring stations that measured an air pollutant in ppb would be discarded. The case of ppb measurements only occurred in monitoring stations for NO_2_, O_3_ and SO_2_, with the impact of the spatial distribution of monitoring stations visible in electronic supplementary material, figure S1.

We excluded further monitoring stations from the dataset based on several criteria indicative of poor data quality. These criteria included stations with two or fewer data points, occurrences of repeated time stamps with differing air pollution concentration measurements, and stations recording all measurements as zero or consistently reporting a single value across all time stamps. To ensure the high quality of data for our model, we removed stations lacking an hourly reading for each hour of the day across their dataset (e.g. at least one measurement for hours 0000−2300) and at least one measurement for each day of the week. Although the exclusion of data based on these criteria may appear extensive, they constituted a minor portion of the total dataset. After cleaning, the dataset still comprised considerable numbers: 2894 stations for NO_2_, 2076 for O_3_, 2924 for PM_10_, 2939 for PM_2_._5_ and 1446 for SO_2_. As a proof of concept in this study, we focused on 2022.

### 3.2. Feature vector

While data concerning a range of phenomena that act as sinks or sources of air pollution could be included in this study, we decided to limit the input data to data sources with global coverage with a consistent schema. The goal was to simplify the model to be created and remove the need to worry about integrating potentially hundreds of datasets from national datasets to achieve global coverage, which we saw as outside this work’s scope in achieving the goals outlined in §1. As such, we focused on four prominent datasets concerning broad phenomena related to air pollution, each containing individual datasets describing particular phenomena. The study includes temporal, meteorological, remote sensing and emissions datasets, with a total of 26 feature vector elements.

*Temporal, five features*. Air pollution concentrations exhibit various cyclical patterns, including diurnal cycles influenced by rush hour traffic for NO_2_ [39] and sunlight hours for O_3_ [40]. Additionally, weekly trends emerge due to the working week’s schedule [41,42]. Seasonal cycles, driven by residential heating, elevate air pollutants during winter months [43,44]. Moreover, winter conditions stabilize atmospheric movements, diminishing air pollution dispersion [45], with specific effects on O_3_ levels due to colder temperatures and reduced sunlight [46]. Consequently, the hour, day of the week, week number and month were incorporated as elements of the feature vector, based on Coordinated Universal Time (UTC) to maintain consistency across different time zones globally. However, utilizing UTC presents challenges, such as misalignment with local times for specific activities, like rush hour. To address this, another feature vector element, the UTC offset, was introduced to account for the difference between the local time and UTC time.

*Meteorological, 11 features*. A range of meteorological phenomena plays a crucial role in air pollution concentrations. The direction and speed of air pollution dispersion are heavily influenced by wind [47]. Temperature can influence air pollution through a range of processes such as temperature inversions [48], O_3_ production [49], alongside direct solar radiation [50]. Temperature can also have impacts indirectly by changing air pollution removal by plants [51]. Rainfall drives key air pollution removal processes, such as wet disposition [52] and wash-off from surfaces [53,54]. Pressure can influence air pollution concentrations, with vertical mixing occurring at higher levels in low-pressure systems dispersing air pollutants [55], alongside the reverse occurring in high-pressure systems [56]. O_3_ production also occurs at higher rates at higher pressures [57]. The boundary layer further influences vertical mixing [58], alongside its height providing the overall atmospheric volume for air pollution to concentrate [58,59].

**Figure 2.**
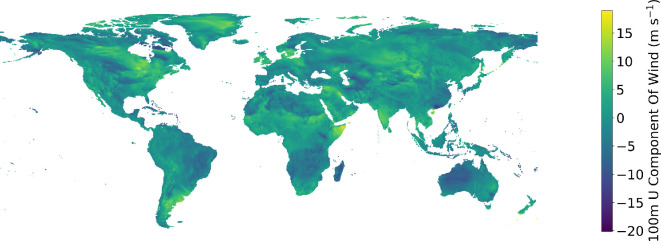
100 m U component of wind from the meteorological dataset family.

Meteorological data were sourced from the ECMWF Reanalysis version 5 (ERA5) dataset [60]. ERA5 provides historical estimates for hundreds of environmental phenomena at locations worldwide, encompassing the entire duration of this study. The dataset was linearly spatially interpolated to approximate values for specific locations required in the study, including the sites of actual monitoring stations used in the model’s training phase or the positions of synthetic air pollution monitoring stations when generating a comprehensive air pollution map across the globe. The meteorological dataset family comprising the features 100 m U component of wind (shown in figure 2), 100 m V component of wind, 10 m U component of wind, 10 m V component of wind, 2 m dewpoint temperature, 2 m temperature, boundary layer height, downward UV radiation at surface, instantaneous 10 m wind gust, surface pressure and total column rain water.

**Figure 3.**
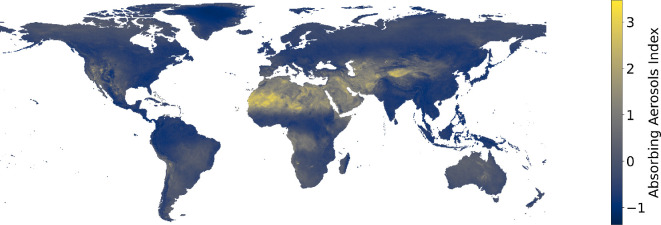
Absorbing aerosol index from Sentinel 5P for the remote sensing dataset family.

*Remote sensing, four features*. The remote sensing data utilized in this study were obtained from the Sentinel 5P platform for the year 2022, where a monthly average for each location was computed to address the data quality issues outlined in §2.1. The selected variables for analysis included NO_2_, O_3_, SO_2_ and the absorbing aerosol index (as shown in figure 3).

*Emissions, six features*. Emissions from various processes are critical in determining the air pollution concentrations within a specific area. The Copernicus Atmosphere Monitoring Service (CAMS) global anthropogenic emissions datasets [61,62] from Emissions of atmospheric Compounds and Compilation of Ancillary Data (ECCAD) [63] were incorporated to characterize emissions of CO, NO_*x*_, non-methane volatile organic compounds (NMVOCs), other VOCs and SO_2_ from the following sectors:

(1) *Refineries*. Refineries refer to the industrial facilities for processing crude oil into products such as gasoline, diesel fuel, etc., producing several different air pollutants [64].
(2) *Ships*. Ships emit NO_*x*_, PM, CO_2_ and VOCs [65], and particularly SO_*x*_ from the marine fuels with high sulfur content [66]. However, there has been a marked reduction in SO_*x*_ as new regulations for sulfur content in marine fuels have emerged recently, substantially reducing emissions [67].
(3) *Fugitives*. Fugitive emissions are unintentional and undesirable emissions, leakage or discharges [68]. While fugitive emissions are smaller than other sources, they represent a measurable factor in air pollution concentrations [69].
(4) *Power generation*. The source of energy greatly influences the emissions from the power sector. If the fuel contains sulfur, then when burned, SO_2_ is produced. Coal, particularly bituminous coal and lignite, contains significant amounts of sulfur [70], with the particular amount depending on the origin of the coal [71]. SO_2_ can also be emitted from crude oil and oil-derived fuels like diesel, heating and bunker oil containing sulfur [72]. Likewise, natural gas contains sulfur, albeit at a much lower content than other fuels [73]. Biomass (wood and crop residues, etc.) can also produce harmful air pollution such as SO_2_ [74].
(5) *Off-road transportation*. This refers to any transportation device not used on public roads. Some of the phenomena described by this sector include railway activities emitting PM, NO_*x*_ and CO_2_ [75], aeroplanes emitting CO_2_, NO_*x*_, SO_2_, PM, unburned hydrocarbons and black carbon [76], alongside agricultural [77], construction [78] and mining equipment [79].
(6) *Road transportation*. Road vehicles exhaust gas air pollutants such as CO, CO_2_, NO_*x*_, SO_2_ [80] and PM air pollution [81].
(7) *Residential*. The residential emissions sector captures residential activities and household sources, including heating [43] and cooking [82].
(8) *Industrial process*. This refers to a range of different activities that release emissions, such as chemical manufacturing [83–85], cement production [86], production of plastics and emissions of VOCs [87].
(9) *Solvent*. Solvents can be used for various processes encompassing manufacturing, printing, automobile and pharmaceutical industries with associated emissions [88].
(10) *Agricultural waste burning*. Waste burning in agriculture can occur for a range of reasons, such as disease control, pest control, crop propagation or crop rotation [89], with implications on air quality [90].
(11) *Solid waste and waste water*. Solid waste refers to emissions from processes such as landfills [91,92] and waste treatment, such as incineration [93]. Wastewater treatment plants also release emissions [94].

**Figure 4.**
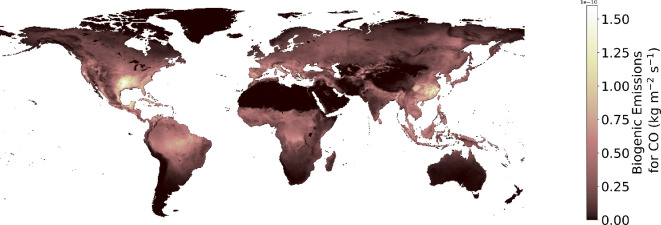
Biogenic emissions example for the emissions dataset family.

In addition to anthropogenic sources of emissions, biogenic emissions of CO are also accounted for using the CAMS Global Biogenic Emissions dataset [62,95,96], which captures emissions from organic matter [97], as shown in figure 4.

*Overview, 26 features*. Across all of the different feature vector datasets, 26 different feature vector elements were included. These 26 feature vectors were chosen as they are provided on a global scale, ensuring complete coverage, enabling models created to be used to predict air pollution concentrations at a global spatial level. The feature vectors used are: *Meteorological*: 100 m U component of wind, 100 m V component of wind, 10 m U component of wind, 10 m V component of wind, 2 m dewpoint temperature, 2 m temperature, boundary layer height, downward UV radiation at surface, instantaneous 10 m wind gust, surface pressure, total column rain water, *Remote Sensing*: Sentinel 5P NO_2_, Sentinel 5P absorbing aerosol index, Sentinel 5P CO, Sentinel 5P O_3_, *Temporal*: week number, month number, day of week number, hour number, UTC offset, *Emissions*: anthropogenic emissions CO, anthropogenic emissions NO*_x_*, anthropogenic emissions NMVOCs, anthropogenic emissions other VOCs, anthropogenic emissions SO_2_, biogenic emissions CO.

## 4. Models

### 4.1. Model design and thinking

LightGBM [98] was chosen as the machine learning approach for the problem, having been used successfully in our previous work in estimating air pollution concentrations across England. LightGBM is a gradient-boosting decision tree (GBDT) algorithm where an ensemble of decision trees are trained in a sequence, with the *n* + 1 decision tree fitting the residuals of the first *n* decision trees, learning the difference between the actual target vector and the weighted sum of predictions of the first *n* decision trees.

Air pollution concentration datasets frequently contain various anomalies and outliers, a challenge that persists across both individual monitoring stations and wider networks [99,100]. This issue is exacerbated in global datasets encompassing multiple monitoring station networks. Consequently, the selection of a model robust against both outliers and anomalies was crucial. LightGBM addresses this challenge effectively through its use of decision trees as the foundational learning mechanism. These decision trees aim to cluster homogeneous data instances, facilitated by the ‘minimum data in leaf’ parameter. This parameter sets a threshold for the smallest number of data instances that constitute a valid leaf, thereby ensuring that the LightGBM algorithm only considers a sufficiently large and homogeneous set of data points for learning and prediction. For example, in the context of air pollution prediction, an anomalous observation—such as an unusually high pollution reading coinciding with high wind speed and remote sensing data, which occurs only once in the dataset—would not lead to the formation of a dedicated leaf. This scenario could arise from a transient pollution source, like a vehicle passing near a monitoring station, resulting in a measurement that inaccurately reflects the broader area’s air quality.

A further benefit of LightGBM over other models is the approach taken to building the model. The approach that LightGBM takes when building the decision trees is to split observations based on the feature vector values, looking for the best possible split regarding information gain and reducing the uncertainty regarding the target vector, grouping homogeneous instances of data points, such as the instances where there is high wind speed at a monitoring station that is measuring low concentration readings for NO_2_. One of the core issues with our air pollution training data is that many data points within the datasets repeat the same information due to the cyclical nature of air pollution measurements, causing a considerable amount of bloat in the datasets. The standard approach to identifying split points within a GBDT is the pre-sorted algorithm where all possible split points are explored, an approach which, in this use case, would be highly costly regarding computation and memory. LightGBM helps tackle this issue by using histograms when performing the splits, where continuous variables are put into discrete bins, changing the computational cost from dependent on the number of data points to the number of discrete bins created.

In this study, we did not implement a dedicated feature selection process before model training, primarily because our chosen machine learning algorithm, LightGBM, inherently performs feature selection during the training process. As a decision-tree-based model, LightGBM splits at features that provide the most information gain or reduction in impurity at each node. Therefore, only the most informative features contribute to the model’s predictions, and uninformative or redundant features are automatically excluded from the final model. For example, even though both 10 and 100 m wind features were included in the input data, their correlation, or multi-collinearity, does not degrade model performance, as LightGBM can effectively handle such relationships without the need for additional pre-processing. Including both features allows the model flexibility without imposing any computational penalty or impacting predictive power, as redundant features are ignored.

Moreover, the model can adapt its feature selection process to different species of air pollutants. For example, while the 10 m wind feature may be more relevant for predicting NO_2_ concentrations, other species like SO_2_ might rely more heavily on features like anthropogenic emissions. The flexibility of the LightGBM model allows it to dynamically adjust the feature importance for each air pollutant, ensuring that the most relevant information is utilized for each specific prediction task without the need for manual intervention or predefined feature subsets.

There are, however, some trade-offs to the use of LightGBM. The feature importances given for the feature vectors via the model will probably be misleading due to multi-collinearity present from similar feature vectors, such as the wind speeds at 10 and 100 m, where the model is able to extract information about air pollution concentrations from either feature. Therefore, during model building, in the split performed, the model would use only the 10 or 100 m component of the wind direction, as they would present the same information gain about the target variable as each other. As the feature importance given by LightGBM is based on the number of times a feature vector is used, the total number of times the two feature vectors are used may be split across the two features, reducing the feature importance given to each one. There are also implications for any sensitivity analysis conducted, as it is possible that we could increase/decrease the 100 m component of wind and there be a misleading change in the air pollution concentration prediction if the model used the 10 m wind component as the split point. Therefore, if feature importance is to be analysed, a more sophisticated method such as SHapley Additive exPlanations (SHAP) [101] needs to be used. SHAP is a game-theory-based approach used in machine learning to explain the contribution of each feature to model predictions. It assigns each feature a ‘SHAP value’ that represents its impact on the prediction, providing a more nuanced understanding of feature importance by accounting for feature interactions and multi-collinearity. Unlike traditional feature importance methods, SHAP ensures that the contributions of all features are fairly distributed, making it a robust tool for model interpretability.

A key consideration during the model design was the choice of the loss function. The loss function represents the error of a given prediction, in this case, quantifying the difference between the actual air pollution concentration and prediction of the model, thereby allowing for comparisons between model variations and subsequent choice of the optimal model configuration. The choice of the loss function in this situation was between the mean absolute error (m.a.e.) and the mean squared error (m.s.e.) [102]. The m.a.e. would help reduce the influence of higher air pollution measurements on the model present due to the known presence of outliers and anomalies within the dataset. However, these high air pollution measurements are of vital interest within the context of air pollution, even if they are potentially erroneous. So, a trade-off of potentially overfitting on these higher values was seen as a worthwhile trade-off, and as such, the m.s.e. was chosen as the loss function. The underlying premise is that 10 µg m^−3^ is more than twice as bad on human health than 5 µg m^−3^, so using the m.s.e. is more appropriate, given the domain in which the model would be used. Supporting the existing literature understanding that there is a nonlinear relationship between the detrimental effect of air pollution concentrations increases and damage to human health and well-being [103,104].

As an air pollution concentration can never measure less than zero, we trained the model on the log transformation of the target vector. We added a small constant of 1 × 10^−7^ to the target vector and then performed the log transformation due to 0 concentration measurements within the dataset. The log transformation and addition ensured that the model would never predict a negative value as the model output, as the reverse transformation of calculating the exponential and subtracting 1 × 10^−7^ was performed on the output. A further hyperparameter explored during model training was L2 regularization [105]. Including L2 regularization helps distribute the weights within the decision tree, encouraging the weights to be closer to 0 but keeping all feature vectors, ensuring that no single feature vector drives predictions, key with the considerable number of feature vectors used.

The framework for choosing the model’s hyperparameters was a randomized grid search of five parameter sets. The parameters we optimized during the randomized search were the L2 regularization and the min data in each leaf already discussed, alongside the number of leaves, the number of trees and the max depth [106]. The number of leaves search space was given the range of 1000 to 4095 [107], with the optimal values being chosen near the centre of this range, validating its choice. The number of trees was controlled via early stopping, where no additional tree would be added after 10 trees had been added without any improvement in the loss function performance. Similarly, the max depth was not limited and left to grow as needed until performance did not improve during training.

Some model parameters were kept constant throughout the search, such as the max bin, kept constant at 63. The max bin refers to the number of discrete bins created for a continuous feature vector. Sixty three was chosen to ensure that a range of different splits during model training could be created while also helping to reduce training time by allowing data to be stored optimally as an int8 data type, with minimal reduction in model accuracy [106]. The boosting type used during training was gradient-based one-side sampling (GOSS) [98]. GOSS is a method of boosting that allows the *n* + 1 decision tree discussed at the start of this section to be trained on a subsample of the data. The subsample of data chosen is the data that has a large gradient, i.e. the data the model has yet to learn well from and a random sample of the small gradient data, helping to reduce the amount of data used drastically, and therefore training time. The trade-off with GOSS is the potential for overfitting when the datasets are small; however, this was not a concern in the context of air pollution dataset used for training in this study.

The data was split randomly into 70% training, 20% validation and 10% test sets. The data was split into sets by the magnitude of the concentrations, based on the UK DAQI [4]. Each index of the DAQI data points was put equally into each set to ensure that potential outliers or anomalous points existed in each of the three datasets; if less than three data points for a particular DAQI band were in the monitoring station data, then all the data points would be put into the training set. Hold-out validation was acceptable due to the considerable dataset size and the minimal gain in overall performance at the cost of considerable additional computational cost [108]. We chose the best parameter set based on the model’s m.s.e. on the validation set across the parameter sets.

As we wanted to allow the possibility of extending the model as new data becomes available, we did not include any feature vector element detailing monitoring station identifiers, such as the name or location where the observation was measured. A further consideration was the lack of inclusion of lags of the air pollution concentration, such as using the concentration at *T* − 1 to estimate the concentration at time *T*. Thereby allowing us to make estimations where there has never been an air pollution measurement. Further, this allows us to mix observations where subsequent observations were treated as independent. This allows the data to be put into a tabular format, where LightGBM has state-of-the-art performance. Together, these elements of temporal and spatial independence of observations allow the creation of a lightweight model where parallel computation is possible of different locations and time points.

### 4.2. Predictive capability

The first experiment conducted during the study explored how well a data-driven supervised machine learning model could learn the relationship between air pollution concentrations and the feature vectors described in §3.2. The *R*^2^ score was used as the evaluation metric during our experiments [109]. The *R*^2^ score embeds our baseline model for comparison (i.e. the null model for the problem), where an *R*^2^ score of 0 represents a model that has predicted the mean for every time point in the series. If an *R*^2^ score is negative, then it represents a model that contributes no utility to estimating air pollution concentrations over the information already contained within the concentration dataset; as such, the threshold for a successful model is seen as one with a positive *R*^2^ score, providing additional explanation for the variability seen in the concentrations over the null model. In this study, we have not used m.a.e., m.s.e. or other associated metrics due to the large differences in orders of magnitude between the concentrations of different air pollutants; for example, SO_2_ has a maximum value of 10 orders of magnitude smaller than O_3_, making comparisons between models that estimate different air pollutants with such metrics inappropriate.

Table 1 shows the number of monitoring stations with a positive *R*^2^ score. It can be seen that the model works well for nearly all of the air pollution monitoring stations included in the study. The reduced performance for SO_2_ can be explained by the limited amount of data, with only 1446 monitoring stations.

**Table 1.** Number of monitoring stations with positive *R*^2^ scores with a model trained on all data. The table shows a baseline set of results for the model’s predictive capability to learn the relationship between the feature and target vector as a comparison for future experiments. The baseline aims to accurately represent the data quality used, highlighting the number of stations with questionable data, such as CAAQM 8171 shown in figure 5a that probably represents incorrect data. The results show that the model can capture the relationship across the monitoring stations while not being limited to performing well in a single continent.

pollutant name	number of stations	Asia	Australia	South America	Africa	Europe	North America	Oceania
NO_2_	2613/2894 (90%)	201/295 (68%)	—	12/12 (100%)	53/55 (96%)	2345/2527 (93%)	2/5 (40%)	—
O_3_	1904/2076 (92%)	187/273 (68%)	—	7/10 (70%)	48/50 (96%)	1658/1739 (95%)	4/4 (100%)	—
PM_10_	2780/2924 (95%)	439/520 (84%)	49/53 (92%)	94/101 (93%)	59/75 (79%)	1779/1804 (99%)	357/368 (97%)	3/3 (100%)
PM_2.5_	2573/2939 (88%)	337/433 (78%)	13/63 (21%)	97/114 (85%)	62/77 (81%)	1071/1127 (95%)	993/1111 (89%)	0/14 (0%)
SO_2_	882/1446 (61%)	193/280 (69%)	—	47/66 (71%)	30/51 (59%)	611/1048 (58%)	1/1 (100%)	—

**Figure 5.**
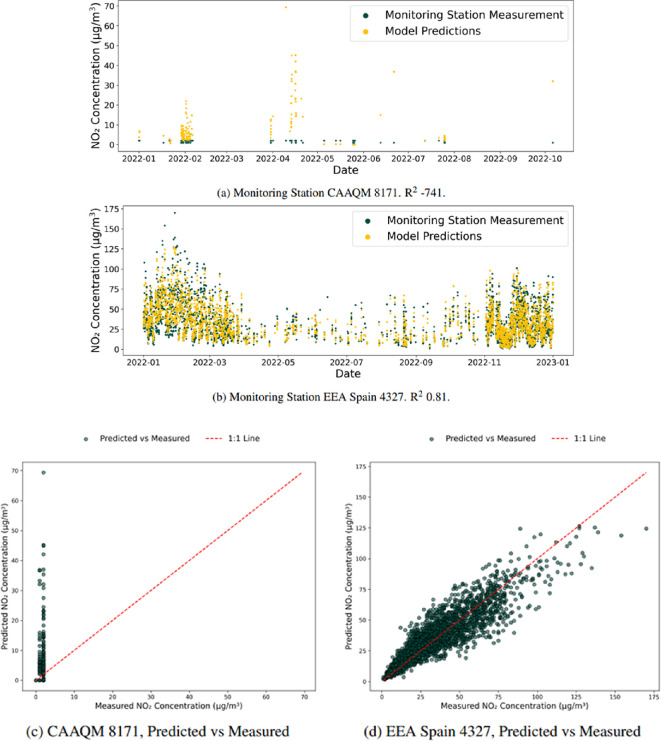
Model predictions for a well-performing (EEA Spain 4327) and a poor-performing (CAAQM 8171) monitoring station during the baseline experiment. While the model does not perform well for CAAQM 8171 (shown in a), looking at the data does raise questions about its validity, with the NO_2_ concentrations having poor precision with only integers recorded and never exceeding 2 µg m^−3^, highly unlikely given the industrial location of Nandesari, India. By contrast, EEA Spain 4327 (shown in b) performs very well, with data that appears accurate. Highlighting the contrast in the quality of data in the dataset used and the importance of performing a baseline experiment rather than simply assuming all monitoring stations represent accurate data. (c) Shows the CAAQM 8171 predicted versus measured values, showing poor agreement with observations, with predicted values deviating substantially from the 1:1 line, probably due to data precision issues. (d) Shows the EEA Spain 4327 predicted vs measured values, showing strong agreement with predicted values closely aligning with observed concentrations, indicating reliable predictions.

Even with the stringent criteria used to subset the dataset (see §3.1), some monitoring stations with questionable data remain in the study. A notable example is the Indian air pollution monitoring station Continuous Ambient Air Quality Monitoring (CAAQM) 8171, as reported in the OpenAQ dataset maintained by the Central Pollution Control Board of India [110]. For this station, our model achieved an exceptionally low *R*^2^ score of −741. Figure 5a displays the station’s measurements, which are recorded as 129 measurements at 2 µg m^−3^, 40 at 1 µg m^−3^ and 2 at 0 µg m^−3^.

The first concern arises from the measurement resolution itself; such coarse quantization immediately raises questions about data quality. The situation is compounded by the station’s location—at longitude 73.1 and latitude 22.4, it corresponds to Nandesari, India, an urban area characterized by major industrial activity. While the reported figures may be accurate as per the official records, they appear inconsistent with the expected air quality levels in such a dynamic environment. This discrepancy underscores the broader issue of data reliability that must be accounted for when analysing our results.

By contrast, figure 5b shows the time series from monitoring station European Environment Agency (EEA) Spain 4327, where the measurements exhibit more variability and complexity. Here, our model successfully captures the temporal trends, yielding an *R*^2^ score of 0.81. This juxtaposition motivates the initial baseline experiments we conduct within the next section.

### 4.3. Estimating missing stations

Section 4.2 shows that a machine learning model framework can learn the relationship between the outlined feature vectors described in §3.2 and air pollution concentrations, successfully estimating air pollution concentrations in a location it has been trained on. However, to create a complete spatial picture of air pollution concentrations, the model must estimate locations where no training data is included. Leave-one-out validation (LOOV) explores the model’s performance in this scenario. LOOV leaves out particular subsets of data from the training set that have some semantic meaning. Semantic meanings within this work refer to data which belong to a single network, country or continent. Each data subset is sequentially removed from the training set, and the model is trained on the remaining data. The excluded data subset is then used to test the model’s predictive capability. This process is repeated until every data subset has been left out once. This method provides an assessment of the model’s ability to generalize to unseen data, simulating the scenario of predicting air pollution concentrations in locations without prior measurements included in the training data. The following experiments indicate model performance when data for particular stations within a network (§4.3.1), a whole country (§4.3.2) or continent (§4.3.3) has been left out.

#### 4.3.1. Within network

**Figure 6.**
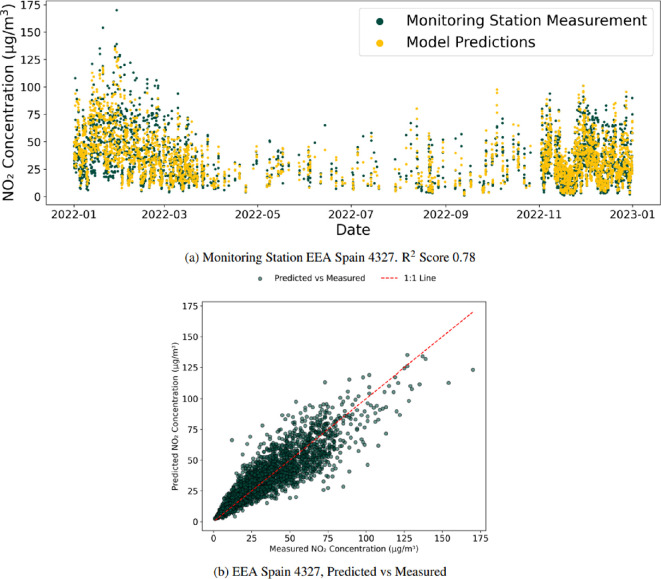
Monitoring station EEA Spain 4327. *R*^2^ score 0.78, for the within network experiment. For EEA Spain 4327, the model still performs well when it has not seen any of the data from this location, with the *R*^2^ reducing by 0.03, as shown in (a). The slight drop in performance highlights that the model can estimate missing locations well in the case of the EEA Spain monitoring network when it has seen some monitoring stations within the network. (b) Shows that the model maintains good agreement with observations despite a slight drop in *R*^2^, showing similar overall trends as in figure 5d.

Within the air pollution concentration datasets are several different monitoring station networks. The definition of what constitutes a monitoring network is undefined due to the range of different stakeholders that establish and maintain the networks. In this sense, the term monitoring station network is used to group together stations that share some characteristics with each other and are owned and managed by a single entity. The first missing station experiment aims to understand the model’s performance when estimating monitoring stations that it has never seen before, but it has seen data from other monitoring stations within the network. A 10-fold LOOV was conducted. Each monitoring network had its monitoring stations split into 10 groups, with a model trained on nine groups and the final group having their measurements estimated and then assessed. As seen in table 2, there is a drop in the model’s performance when estimating monitoring stations it has not seen before. Still, there is a signal that the model works as intended and can estimate the air pollution concentrations. For example, figure 6 shows monitoring stations EEA Spain 4327 for this experiment, which experienced a performance drop of 0.03 in *R*^2^ score compared with the baseline.

**Table 2.** Number of monitoring stations with positive *R*^2^ scores with a model trained on some stations in the same monitoring network. The first missing station experiment explored the model’s performance when estimating a given station when no data concerning that station has been seen. The model has only seen data from a portion of the target monitoring station network, with no data from the particular monitoring station of interest. It can be seen that the relationship between continents broadly remains the same as table 1. However, the number of stations is meaningfully reduced. Of note is that the results between air pollutants are to be expected with O_3_ performing the best, being driven primarily by meteorological conditions, the highest spatial and temporal resolution and quality feature vector used by the model.

pollutant name	number of stations	Asia	Australia	South America	Africa	Europe	North America	Oceania
NO_2_	860/2894 (30%)	37/295 (13%)	—	1/12 (8%)	6/55 (11%)	815/2527 (32%)	1/5 (20%)	—
O_3_	1495/2076 (72%)	50/273 (18%)	—	3/10 (30%)	24/50 (48%)	1418/1739 (82%)	0/4 (0%)	—
PM_10_	388/2924 (13%)	85/520 (16%)	0/53 (0%)	3/101 (3%)	1/75 (1%)	286/1804 (16%)	13/368 (4%)	0/3 (0%)
PM_2.5_	873/2939 (30%)	177/433 (41%)	3/63 (5%)	29/114 (25%)	5/77 (6%)	457/1127 (41%)	202/1111 (18%)	0/14 (0%)
SO_2_	6/1446 (0%)	2/280 (1%)	—	0/66 (0%)	0/51 (0%)	4/1048 (0%)	0/1 (0%)	—

#### 4.3.2. Between countries

**Figure 7.**
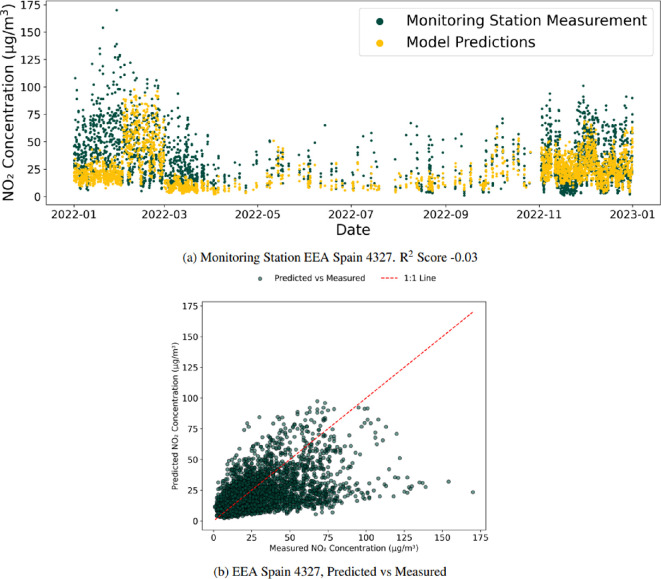
Monitoring station EEA Spain 4327. *R*^2^ score −0.03, for the between-country experiment. When the model has not seen any data for Spain, the estimation for EEA Spain 4327 performs poorly, with a negative *R*^2^ score, as shown in (a), representing a prediction worse than the mean of the time series. (b) Shows the poor performance when the model is trained without any data from Spain, with predictions showing little agreement with observations, frequently deviating from the 1 : 1 line.

The second missing station experiment performed LOOV based on the country of the monitoring stations. In this case, the experiment aims to understand the model’s performance when estimating a country’s air pollution concentrations when it has not seen any data concerning the country, exploring, for example, the ability to estimate Spain’s monitoring station measurements when only stations outside Spain have been used for model training. Figure 7 shows the same monitoring station of EEA Spain 4327 as before; however, the *R*^2^ has now dropped considerably to −0.03, representing a model that is estimating worse than the average of the time series. Table 3 shows the results for this experiment.

**Table 3.** Number of monitoring stations with positive *R*^2^ scores with a model trained on stations only outside the target country. The between-country experiment repeats the same process as the within-network experiment shown in table 2 to explore the possibility of estimating given countries’ monitoring stations data without any in-country data being seen by the model. It can be seen that the model performance drops considerably. However, there is still a positive signal for each air pollutant other than SO_2_.

pollutant name	number of stations	Asia	Australia	South America	Africa	Europe	North America	Oceania
NO_2_	356/2894 (12%)	4/295 (1%)	—	0/12 (0%)	0/55 (0%)	352/2527 (14%)	0/5 (0%)	—
O_3_	1242/2076 (60%)	22/273 (8%)	—	0/10 (0%)	15/50 (30%)	1205/1739 (69%)	0/4 (0%)	—
PM_10_	173/2924 (6%)	6/520 (1%)	0/53 (0%)	0/101 (0%)	0/75 (0%)	167/1804 (9%)	0/368 (0%)	0/3 (0%)
PM_2.5_	420/2939 (14%)	109/433 (25%)	2/63 (3%)	0/114 (0%)	0/77 (0%)	273/1127 (24%)	36/1111 (3%)	0/14 (0%)
SO_2_	9/1446 (1%)	0/280 (0%)	—	0/66 (0%)	0/51 (0%)	9/1048 (1%)	0/1 (0%)	—

#### 4.3.3. Between continents

**Figure 8.**
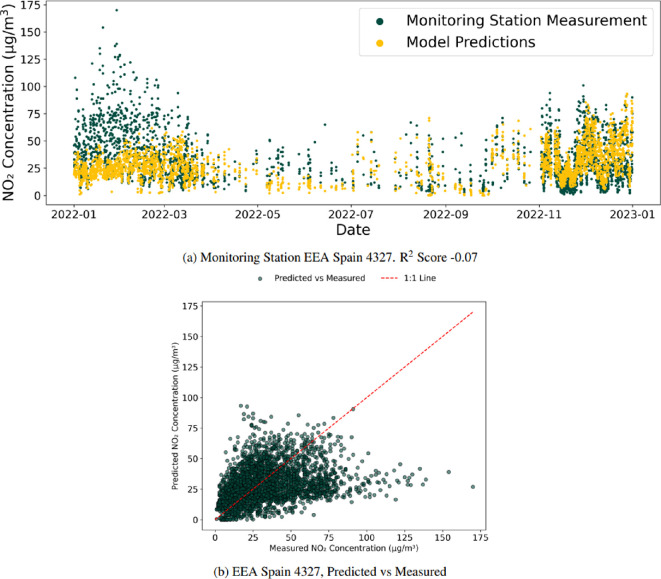
Monitoring station EEA Spain 4327. *R*^2^ score −0.07 for the between-continent experiment. The model’s performance for this station remains broadly the same, with a drop of 0.04, highlighting that for EEA Spain 4327, the drop in performance occurs from not having any data in the country. Still, data further removed do not further meaningfully reduce the performance, as shown in (a). Further, the poor performance of the model can be seen in (b) where there is little agreement with observations and points again frequently deviating from the 1 : 1 line.

The final missing station experiment repeated the same process as §4.3.2 but this time with continents, aiming to determine the ability to estimate one continent’s monitoring station’s measurements when trained only on data from outside that continent. For example, estimating Europe’s air pollution concentration data from a model only trained on data from North and South America, Asia, Australia and Oceania. An example from this experiment is shown in figure 8 for monitoring station EEA Spain 4327, where the *R*^2^ performs marginally worse than the between-countries missing station experiment discussed before. Table 4 shows the results for this experiment.

**Table 4.** Number of monitoring stations with positive *R*^2^ scores with a model trained on stations only outside the target continent. The between-continent experiment results show a clear drop in performance compared with the between-country experiment, highlighting the difference between air pollution concentrations between countries and the need for similar countries to be used to train the model to predict concentrations accurately.

pollutant name	number of stations	Asia	Australia	South America	Africa	Europe	North America	Oceania
NO_2_	154/2894 (5%)	7/295 (2%)	—	0/12 (0%)	0/55 (0%)	147/2527 (6%)	0/5 (0%)	—
O_3_	78/2076 (4%)	18/273 (7%)	—	0/10 (0%)	1/50 (2%)	59/1739 (3%)	0/4 (0%)	—
PM_10_	16/2924 (1%)	14/520 (3%)	0/53 (0%)	0/101 (0%)	0/75 (0%)	1/1804 (0%)	1/368 (0%)	0/3 (0%)
PM_2.5_	8/2939 (0%)	2/433 (0%)	1/63 (2%)	0/114 (0%)	0/77 (0%)	3/1127 (0%)	2/1111 (0%)	0/14 (0%)
SO_2_	5/1446 (0%)	0/280 (0%)	—	0/66 (0%)	0/51 (0%)	5/1048 (0%)	0/1 (0%)	—

### 4.4. Overall trends

**Figure 9.**
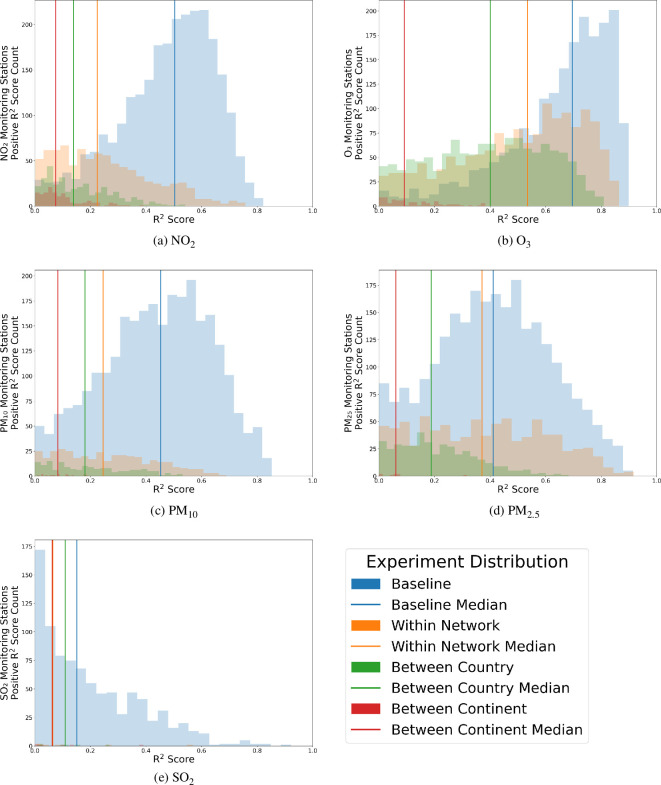
Positive *R*^2^ score distribution for each experiment for each air pollutant. The distribution broadly follows the same trend of the reduction in magnitude and shift to the left with each experiment, except SO_2_, probably driven by the minimal number of monitoring stations available. The distributions suggest that the model framework is robust to different air pollutants. Performance for each primarily depends on the quality of the feature vector describing the phenomena driving a given air pollutant, with O_3_ performing the best while also having its driving phenomena, meteorological conditions, having the highest quality feature vector data, with unique data points spatially and temporally.

Figure 9 shows the overall trends of the different *R*^2^ scores for all monitoring stations across each air pollutant. The trend for the overall distribution is similar to the one seen for monitoring station EEA Spain 4327 for NO_2_. There appears to be a noticeable drop from the baseline to the within-network missing station experiment, with a considerable drop in performance during the between-country experiment, with poor performance maintained in the between-continent missing stations experiment. These initial results suggest that the current iteration of the approach works mainly for estimating air pollution in locations with similar air pollution concentrations, highlighted by the stark drop in performance when a given country’s air pollution measurements are excluded from training. While there is an apparent use of the model in estimating air pollution concentration in countries with a monitoring station, understanding why the model works in this situation is paramount and is what the next section tackles.

## 5. Empowering stakeholders: prediction intervals

As highlighted in §4, the modelling approach does have merit, performing well for some monitoring stations, providing an initial signal that a robust and accurate global air pollution model driven by supervised machine learning can be achieved. However, there are locations in which the model could perform better, particularly in the context of large geographic areas where there are no monitoring stations. In some of these locations, the model has seen similar data before and performs well; in other cases, it may not. To empower stakeholders, we have also created auxiliary models, using the same model framework discussed so far to create a prediction interval for all the predictions the model has made. The prediction interval measures the model’s uncertainty when predicting a location, allowing stakeholders to use or discard data points depending on their risk appetite. For example, it could be the case that a stakeholder requires a small prediction interval size in the problem domain of human health assessments. In contrast, a larger prediction interval might be more acceptable in raising awareness of air pollution in a local community. Due to the range of potential use cases, we cannot prescribe what is acceptable or not and so provide the prediction intervals to empower stakeholders to make their own judgements.

**Figure 10.**
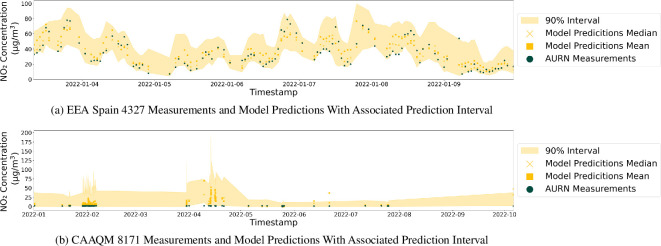
Prediction intervals for a well-performing (EEA Spain 4327) and a poor-performing (CAAQM 8171) monitoring station. As seen in §4, there are some situations in which the model does not work robustly. For all predictions, a 90% prediction interval is provided to ensure any downstream users are aware of model uncertainty for a given prediction. The interval empowers stakeholders to identify and proceed in their desired manner, depending on whether the interval is seen as too large or questionable for a given use case.

Prediction intervals aim to quantify and communicate to downstream users the uncertainty associated with each prediction, helping to ensure that decision-makers understand the broader context of the prediction outside of the point estimate. The prediction interval range helps to highlight situations where the model is making predictions outside of scenarios it has seen before. The prediction interval gives an interval within which a future observation will fall with a certain probability, accounting for the variability of the regression function and the individual observation. Quantile regression is used to create the prediction interval, achieved within the same model framework discussed so far, the difference being that rather than the model being trained to estimate the mean of the air pollution concentration training data, the model is trained to estimate a particular quantile. The three new models created estimate the 0.05, 0.5 and 0.95 quantiles, with the range between the 0.05 and 0.95 quantiles providing the 90% prediction interval. Figure 10a shows the prediction interval for the EEA Spain 4327 monitoring station for the first week of 2022, helping to provide user confidence in the estimations at different data points. As can be seen in figure 10a, the prediction interval stays consistent and broadly smooth, following the overall trend of the data. By contrast, the prediction interval in figure 10b for the CAAQM 8171 monitoring station further highlights the station’s unrealistic air pollution readings. Its irregular pattern suggests that its air pollution measurements do not form the cyclic temporal trend that would be expected, resulting in an erratic prediction interval. Stakeholders can exploit this supporting data to make informed decisions concerning the data used for their study.

Prediction interval coverage probability was used to validate the quantile regression models created. As before with the regression models, the data was split into a 70% training set, 20% validation set and 10% test set following the process outlined in §4.1. Table 5 shows the percentage of data points within the corresponding estimations prediction interval. The consistent coverage of 90% by the training sets across all air pollutants highlights that the quantile regression can learn the 0.05 and 0.95 quantiles accurately. The small drop in performance for the validation set percentage is expected when analysing coverage by data that the model has never seen before and is close to the target of 90%, particularly within the context of the dataset containing questionable data like that of CAAQM 8171. The test set coverage percentage, similar in value to the validation set coverage percentage, further highlights the robustness of the approach.

**Table 5.** Prediction interval coverage probability (PICP) for all air pollutants. The table shows the results of the PICP validation for the quantile regression models. The validation aims to determine the number of data points within an estimations prediction interval, with a target coverage probability of 90%. The results highlight that the quantile regression models are robust and perform to expected tolerance within the context of the problem domain, with 90% coverage for data the models were trained on and a small drop for both the validation and test set, showing consistent performance across unseen data.

air pollutant	train set PICP	validation set PICP	test set PICP
NO_2_	90.1%	82.1%	82.5%
O_3_	90.0%	83.4%	83.2%
PM_10_	90.4%	89.0%	89.0%
PM_2.5_	90.1%	88.7%	88.7%
SO_2_	90.3%	84.0%	84.5%

## 6. Understanding model performance

**Figure 11.**
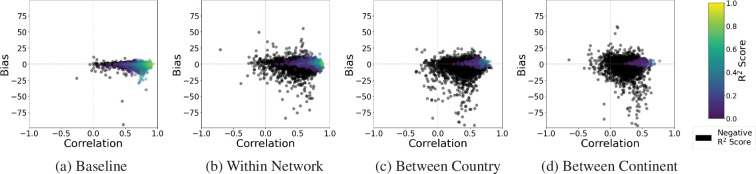
Bias versus correlation for NO_2_ for each of the experiments. It can be seen that the correlation has a weaker relationship with a positive *R*^2^ score than the bias does, with data points for nearly all values of correlation between 0 and 1 in figure 9a having a positive *R*^2^ score, with data points deviating from a 0 bias having a negative *R*^2^ score. This relationship highlights that a high-magnitude bias is driving the poor model performance for some monitoring stations.

While the *R*^2^ provides a good indication of the model’s performance for a given monitoring station’s prediction, the bias and correlation of the prediction can provide further insight into the model’s performance. Bias represents the average difference between the monitoring station measurements and the model predictions, providing a metric for the overall tendency of the modelled values to be higher or lower than the observations. Correlation quantifies the linear relationship between the monitoring station measurements and the model predictions, reflecting how well the model captures the temporal variations, with the Pearson correlation coefficient being used in this study. For EEA Spain 4327, the correlation and bias for the baseline are 0.9 and 0.64, respectively, with 0.89 and 0.75 for the within-network experiment, and finally, 0.44 correlation and −8.8 bias for the between-country missing stations experiment where the *R*^2^ dropped below 0. Reanalysing the EEA Spain 4327 monitoring station with these statistics highlights that the low *R*^2^ is driven primarily by bias rather than the correlation, indicating the model can capture the overall trend of the air pollution concentrations but struggles with the assignment of particular values unless air pollution measurements in a similar location to the one being estimated have been seen. The overall trends across all of the monitoring stations can be seen similarly, with figure 11 showing the correlation against the bias for each monitoring station, with the points coloured by their *R*^2^ score for NO_2_. Across the monitoring stations, as the experiments progress from the baseline, it can be seen that the points at the 0 bias are the points with a positive *R*^2^ score across a range of different correlations. In the within network experiment, some monitoring station predictions exhibit a large magnitude bias with a negative *R*^2^ score, which is further highlighted in the between country and continent experiment. Electronic supplementary material, figure S2 shows the bias-correlation plots for the other air pollutants.

Table 6 shows the 90% interquartile range for the correlation, bias and mean absolute percentage error (MAPE) [111] for all the air pollutants studied, where it can be seen more clearly that the correlation remains constant when compared with the notable changes in the bias and MAPE, further supporting the idea that the increase in bias magnitude is what causes the reduction in *R*^2^. Further supported when analysing the case of monitoring station EEA Spain 4327, shown in figures 5b–8–8, the process that appears to be followed is that the inclusion of data in a location that is to be estimated appears to ‘ground’ the time series prediction, with the overall trend and correlation of the time series remaining broadly constant but the bias changing, driving changes in *R*^2^ score.

**Table 6.** The 90% interquartile range (IQR) for correlation, bias and mean absolute percentage error (MAPE) for each experiment for each air pollutant. The IQR for the correlation, bias and MAPE for each air pollutant follows the same trend shown in figure 11 for NO_2_. The IQR for each of the air pollutants for each of the experiments broadly remains the same, apart from a slight reduction in the last experiment, between continent when all of the correlation values shift downwards, causing a reduction in the IQR. By contrast, the bias doubles in magnitude between the baseline and within experiments and further increases in subsequent experiments. Similarly, the MAPE values show a marked increase, particularly in the later experiments, highlighting the growing discrepancy between predictions and actual values. These increases in bias and MAPE are the primary drivers of the reductions in model performance, as discussed in §4.

air pollutant	correlation baseline	correlation within network	correlation between country	correlation between continent	bias (µg m^−3^) within network	bias (µg m^−3^) within network	bias (µg m^−3^) between country	bias (µg m^−3^) between continent	MAPE (%) baseline	MAPE (%) within network	MAPE (%) between country	MAPE (%) between continent
NO_2_	0.33	0.52	0.49	0.38	6.10	17.00	20.82	23.55	41.45	148.27	155.60	254.86
O_3_	0.31	0.48	0.53	0.49	6.66	23.48	27.80	35.32	56.59	129.28	189.29	171.06
PM_2.5_	0.45	0.58	0.57	0.40	4.66	7.61	13.09	19.57	55.09	74.66	117.57	200.17
PM_10_	0.40	0.52	0.62	0.35	8.54	18.62	35.41	41.90	42.04	67.82	111.71	194.39
SO_2_	0.60	0.45	0.39	0.36	4.06	9.22	15.62	21.94	71.04	227.88	282.14	1257.75

## 7. Improving model performance

**Figure 12.**
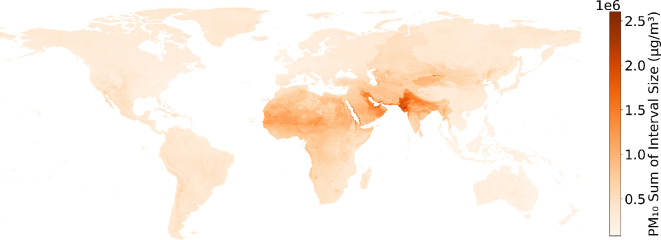
Prediction interval size sum for PM_10_ for 2022. The size of the prediction interval sum across 2022 highlights the locations the model is most uncertain about, providing locations spatially for future monitoring station placement. In the case of PM_10_, it can be seen that Africa, the Middle East and some parts of Western Asia are where the model has the highest uncertainty. Other air pollutants are shown in electronic supplementary material, figure S3.

As highlighted before, acquiring more air pollution concentration data is necessary to reduce the bias experienced when estimating locations dissimilar to previously seen locations. The size of the prediction interval discussed in §5 denoted the predictions with the greatest uncertainty, highlighting locations of particular interest to the model to reduce uncertainty and the placement of future monitoring stations. Figure 12 shows the locations across 2022 that had the largest interval size, providing the locations that would probably benefit from additional data, either in regard to a feature vector dataset describing the phenomena driving air pollution in those locations or simply measurements of air pollution.

## 8. Model interpretation using SHAP

Understanding the decision-making process of machine learning models is crucial, especially in domains such as air pollution, where actionable insights drive policy-making and public health interventions. This section describes the work conducted using SHAP [101] to better understand the relationship between the model’s predictions and the feature vectors, ensuring that the predictions are interpretable and transparent, subsequently improving confidence in the model proposed.

SHAP is a unified framework for interpreting machine learning models. It leverages concepts from cooperative game theory, assigning each feature a ‘SHAP value’ that quantifies its contribution to the model’s prediction. SHAP values provide a consistent method to attribute importance to features, accounting for interactions between them. This approach ensures fairness by considering all possible combinations of features, which is critical in high-dimensional datasets like the one used in this study.

**Figure 13.**
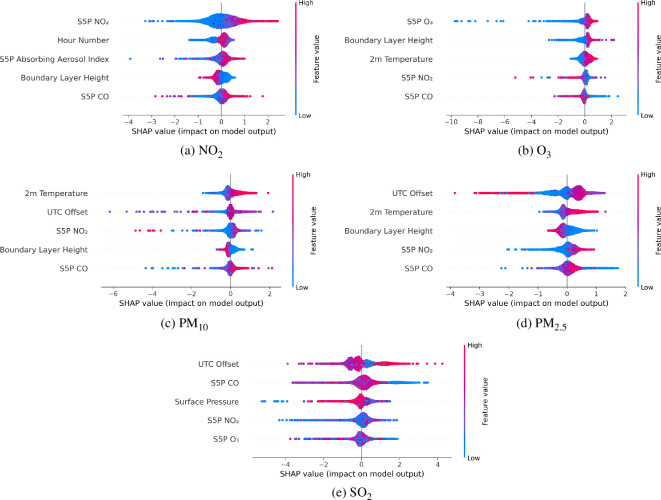
SHAP summary plots for all air pollution models across multiple monitoring stations. Each subplot highlights the SHAP values, indicating the magnitude and direction of each feature’s impact on the model predictions. The SHAP summary plot visualizes the distribution of SHAP values for each feature, ranked by their importance. Each point represents a single data point, and the position along the *x*-axis corresponds to the magnitude and direction of the feature’s impact on the model’s predictions. The colour gradient (from blue to red) indicates the feature’s value, allowing an assessment of whether higher or lower feature values drive predictions in a particular direction.

Figure 13 presents the SHAP summary plots for each air pollutant studied across multiple different monitoring stations. The SHAP summary plot visualizes the distribution of SHAP values for each feature, ranked according to their mean absolute SHAP value. This means that the feature with the highest average impact on the model prediction, regardless of direction (positive or negative), is placed at the top of the plot. Each point represents a single data point, and the position along the *x*-axis corresponds to the magnitude and direction of the feature’s impact on the model’s output. The colour gradient (from blue to red) indicates the feature’s value, allowing an assessment of whether higher or lower feature values drive predictions in a particular direction.

Each subplot in figure 13 highlights the five most influential features driving the model’s predictions for each air pollutant.

Figure 13a shows the summary plot for NO_2_, which identifies the coarse Sentinel 5P NO_2_ measurements as the most critical feature for predicting hourly NO_2_ concentrations in this model. Similarly, the Sentinel 5P measurement for the absorbing aerosol index and CO are features that have a critical influence on model predictions. The emergence of Sentinel 5P NO_2_ measurements as the most influential feature provides confidence that the model is using sensible features, given that it is the only feature vector that directly measures NO_2_ concentrations. The second most influential feature, the hour of the day, aligns well with established scientific understanding of air pollution dynamics, as rush hour traffic has been linked to elevated NO_2_ concentrations [112]. Further, variations in boundary layer height alter the mixing volume, thereby influencing pollutant concentrations [59].

Figure 13b shows the summary plot for O_3_, which again identifies Sentinel 5P measurements as the most critical feature, this time highlighting the O_3_ measurements as the most influential. Additionally, coarse measurements from Sentinel 5P for NO_2_ and CO are also relevant, as both are key precursors in O_3_ formation through atmospheric chemical reactions [50]. Other crucial features include the boundary layer height and 2 m temperature, both of which are well-known to influence O_3_ dynamics. Specifically, higher temperatures and a stable boundary layer height are conducive to photochemical reactions that result in increased O_3_ production [49,59]. These findings align with established scientific understanding of O_3_ dynamics, providing confidence in the model’s ability to capture the key factors that drive O_3_ concentrations.

Figure 13c shows the summary plot for PM_10_. The 2 m temperature is identified as the most critical feature, which aligns with previous literature concerning the influence of temperature on particulate matter dynamics [113,114]. Other important features include the UTC offset, which captures temporal variations in human activity, such as rush hour, during which time increased brake pad use increases particular matter emissions [112]. The boundary layer height is another key feature, as it directly impacts the vertical mixing of air pollutants [59]. The same selection of key features is shown for PM_2.5_ in figure 13d with slight changes in the ordering. These findings align with established scientific understanding of particulate matter sources and behaviour.

Figure 13e shows the summary plot for SO_2_. UTC offset is identified as the most influential feature, which captures temporal variations linked to industrial emissions or other time-dependent processes [115]. Sentinel 5P measurements are crucial to the prediction of SO_2_, as seen in the previous summary plots. Surface pressure is also identified as an important feature, which is closely related to meteorological conditions, such as temperature, humidity and atmospheric stability, which affect the dispersion, transport and chemical transformation of SO_2_ in the atmosphere [116].

The SHAP summary plots have provided valuable insights into the factors driving the model’s predictions for air pollutant concentrations. By leveraging SHAP’s ability to attribute feature importance consistently and transparently, we are able to validate the alignment of model behaviour with established scientific understanding of air pollution dynamics. These findings demonstrate that the models are making predictions based on phenomena that are known to correlate with air pollution concentrations.

**Figure 14.**
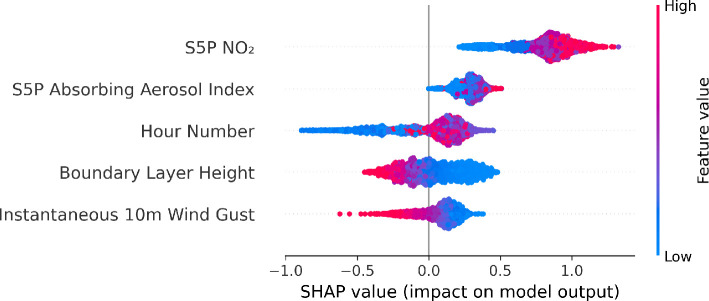
SHAP summary plot for EEA Spain 4327 monitoring station for NO_2_. The five most influential features driving the model’s predictions for NO_2_ pollution concentrations at the EEA Spain 4327 monitoring station are shown.

SHAP summary plots can also be applied to different subsets of the data, such as a single monitoring station. Figure 14 shows the summary plot for the air pollution monitoring station EEA Spain 4327 for NO_2_. For this monitoring station, the most crucial features align with those across all monitoring stations for NO_2_, as seen in figure 13a. However, Sentinel 5P CO has been replaced by a variable describing wind, highlighting that while broad trends remain the same, some key features for individual monitoring stations may change.

**Figure 15.**
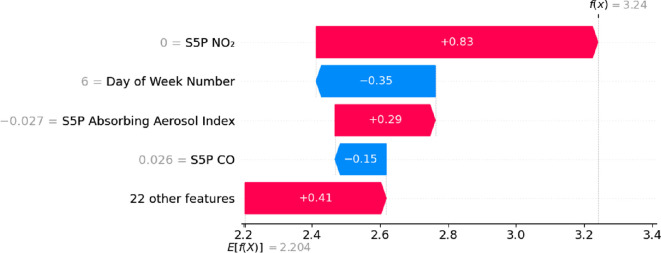
SHAP waterfall plot for EEA Spain 4327 for a single NO_2_ prediction. The waterfall plot explains the individual prediction for the monitoring station EEA Spain 4327 median magnitude data point by breaking down the contribution of each feature to the model’s prediction. As described in §4.1, the raw air pollution concentrations have been transformed into a logarithmic space. The prediction made by the model for this data point is 25.53 µg m^−3^, given by the exponential of *f*(*x*), 3.24. The base value, representing the mean prediction across the dataset, is 9.06 µg m^−3^ (*E*[*f*(*x*)], 2.204). Key features contributing to the prediction include Sentinel 5P NO_2_ (+2.29 µg m^−3^), the day of the week number (+0.7 µg m^−3^), Sentinel 5P absorbing aerosol index (+1.34 µg m^−3^) and Sentinel 5P CO (+0.86 µg m^−3^). The 22 other features have a combined contribution of 1.51 µg m^−3^. Of note is that in this instance, the value of the S5P NO_2_ feature vector is 0.000136 and appears as zero as an artefact of the plotting used by SHAP.

In addition to subsets of the overall data, SHAP can also be used to analyse feature contributions for individual model predictions using a waterfall plot, as shown in figure 15. The *f*(*x*), *E*[*f*(*x*)] and the contribution values in figure 15 are within the logarithmic space due to the transformation of the raw data points, discussed in §4.1. Reversing this transformation provides the value in µg m^−3^. The predicted value for the concentration for this data point is 25.53 µg m^−3^, calculated as the exponential of 3.24 (denoted by *f*(*x*)) and subsequent subtraction of 0.0000001. The waterfall plot also provides the base value shown as *E*[*f*(*X*)] = 2.204 (9.06 µg m^−3^), which represents the model’s expected prediction across the entire dataset, the mean prediction when no feature information is considered. Each of the bars then denotes each feature’s contribution to this prediction, with red bars denoting a positive contribution that increases the concentration predicted and blue negative contributions—the magnitude of their contribution orders the bars, again in logarithmic space. The sum of the base value and all these feature contribution values results in the model’s prediction for this instance. The SHAP waterfall plot is useful for understanding model behaviour at the individual data point level, enabling practitioners to interpret how specific features influence predictions in a transparent and actionable way, allowing for a method of improving stakeholder confidence in predictions.

## 9. Research data output

**Figure 16.**
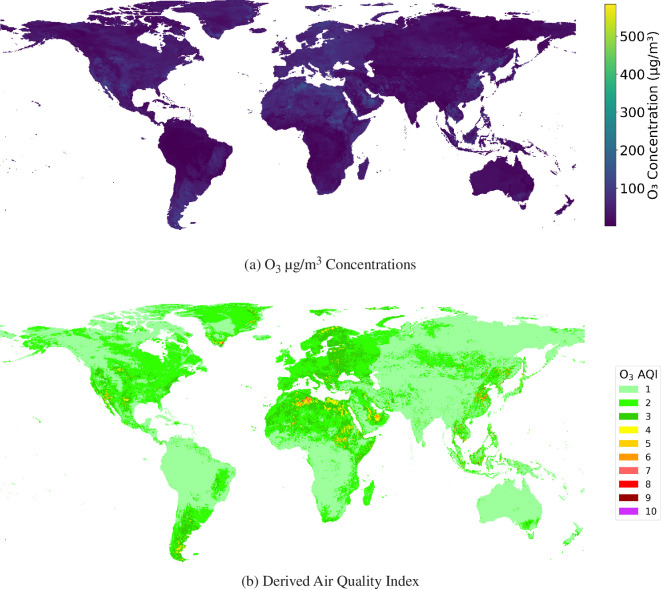
Air pollution maps for O_3_ at 8.00 on 2 July 2022 both in (a) concentrations and (b) Air Quality Index. The figures show the spatial resolution of the model outputs for a single hour, highlighting the locations with high concentrations of O_3_.

**Figure 17.**
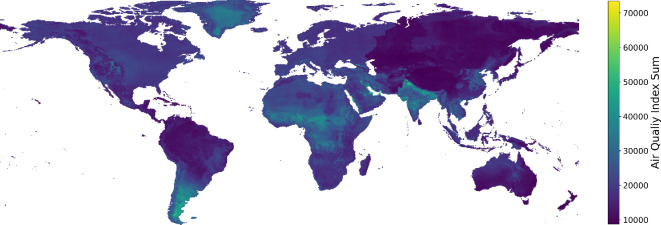
Annual air pollution map for Air Quality Index summation. Each of the individual air pollution maps for each hour shown in figure 16 can be aggregated to the temporal resolution is desired, such as the annual level, to support more strategic decision-making, rather than looking at a potential single outlier peak.

As part of this work, we are providing hourly 0.25° global air pollution concentration data for NO_2_, O_3_, PM_10_, PM_2_._5_ and SO_2_, supporting various downstream assessments which can now be conducted using the high temporal and spatial resolution the data-driven supervised machine learning model provides. The raw concentrations of the air pollutants, shown in figure 16, highlight the differences in concentrations at the hourly level, helpful for scientific activities. However, the concentrations can also be used for public dissemination, such as the Air Quality Index calculated from the concentrations, shown in figure 17.

**Figure 18.**
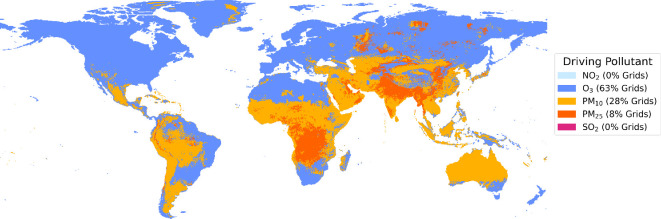
Annual air pollution map for driving Air Quality Subindex. Further to the concentrations map for the annual level shown in figure 17, further analysis can be conducted to understand which air pollutant is driving the poor air quality in different regions, helping to design targeted interventions to the most pressing problems.

Alongside individual hours concentrations being analysed, more long-term analysis can be conducted, such as understanding across the year 2022 the air pollutant that is driving the poor air quality in a given location, shown in figure 18. Understanding the air pollutants driving poor air quality helps to provide a basis for designing interventions, with the interventions for NO_2_ being substantially different from the interventions to reduce SO_2_ concentrations.

## 10. Discussion

The findings presented in §4 offer a comprehensive assessment of the model’s efficacy under diverse conditions, elucidating its capacity to discern the intricate relationship between features and the target vector, while also delineating its constraints in predicting air pollution levels in unmonitored locations. The experimental outcomes reveal that the model exhibits commendable accuracy within known monitoring station networks, where it has previously encountered data. However, its predictive accuracy diminishes for countries without prior data exposure, and this decline is more pronounced for entirely new continents. This gradient in performance underscores the model’s dependency on prior knowledge for optimal prediction accuracy.

While the *R*^2^ score offers a general measure of the model’s performance, an examination of bias and correlation within the results elucidates that the bias is a significant factor in suboptimal model outcomes. The observed strong correlation suggests the model’s proficiency in capturing the general trend of the air pollution concentration time series, yet it encounters difficulties in accurately determining the precise magnitudes. Notably, exposure to training data from the specific location in question markedly mitigates the model’s bias, thereby enhancing the *R*^2^ score. This improvement underscores the indispensability of real-world air pollution measurements to refine model accuracy. Furthermore, leveraging model uncertainty to identify locations of greatest prediction uncertainty illuminates where additional data gathering would yield the most value.

This study introduces a first step towards the development of a lightweight global air pollution model, leveraging a data-driven, supervised machine learning framework. It represents the first endeavour to predict air pollution with such a high degree of spatial and temporal precision, demonstrating the scalable potential of machine learning in addressing complex environmental challenges.

Given the model’s linear computational complexity, there is clear future research that can be conducted to derive further benefits from the model framework proposed within this work. The extension of the method to make predictions at a minute temporal level is straightforward, and similarly, increasing the spatial resolution grid size to 100 m^2^ is trivial, with manageable computational costs, unlike other current air pollution models within the literature that are computationally expensive to operate as discussed in §2.2. Additionally, the encoding of the temporal aspect into a tabular format facilitates a substantial acceleration through parallelization. Each time step and grid within the estimation is independent of one another, enabling the simultaneous calculation of all time steps and grids. This approach yields a significant speed-up over traditional forecasting methods, whether machine-learning or physics-based, that rely on lags from previous time steps, or neighbouring locations spatially.

The model developed in this work is not limited to reconstructing historical air pollution data; it can also be extended to explore hypothetical scenarios and counterfactuals. For instance, the model can simulate potential changes in air pollution concentrations under different emission scenarios, such as evaluating the impact of a specific percentage increase in anthropogenic emissions or changes in meteorological conditions. This flexibility allows users to investigate ’what-if’ scenarios, providing valuable insights into the potential consequences of future policy interventions or environmental changes.

Future enhancements to this research can be broadly categorized into two avenues: data refinement and model enhancement. An immediate next step involves a pre-processing stage for the input data. As illustrated in figure 5a, the quality of data from some monitoring stations appears dubious. Since the model currently does not distinguish between high- and low-quality data during training, any inaccuracies inherently affect model performance. By identifying and addressing anomalies and outliers, either through exclusion or special treatment, model accuracy is expected to improve. Enhancing the feature vector by incorporating additional variables, such as those related to the transportation sector, could further refine the model’s performance.

Moreover, augmenting the model to integrate regional datasets—available in certain areas like North America but not in others like Asia—would ensure the maximal utility of available data. The present model’s requirement for a uniform set of feature vectors across all data points restricts the use of diverse datasets, often resulting in reliance on data with limited spatial and temporal resolution, such as the monthly emissions data employed in this study.

In conclusion, this work plays a pivotal role for a wide array of stakeholders. By providing prediction intervals alongside the predictions themselves, we equip stakeholders with the means to make informed decisions based on their specific risk tolerance, thereby facilitating a spectrum of new applications.

## Data Availability

All the data and code produced during the study is available via Dryad [117]. The air pollution concentration dataset is accessed via OpenAQ Homepage. Sentinel 5P data is accessed via Google Earth Engine Sentinel 5p Catalogue. Meteorology data is accessed via ECMWF ERA5 ECMWF ERA5 Data Repository. Emissions data is accessed via the ECCAD Global Emissions Dataset ECCAD Homepage. Supplementary material is available online [118].

## Footnotes

^1^
For a global view of air pollution monitoring, visit the OpenAQ Global Air Pollution Monitoring Map: https://explore.openaq.org/.

^2^
UK Automatic Urban and Rural Network (AURN) Interactive Map.

^3^
Purple Air Interactive Map.

^4^
GEOS-Chem Hardware Requirements.
